# Novel Combination of Irreversible Electroporation and Allogenic Chimeric Antigen Receptor T-Cell Therapy Synergizes Therapeutic Outcomes in a Preclinical Human Pancreatic Cancer Mouse Model

**DOI:** 10.34133/research.1105

**Published:** 2026-02-02

**Authors:** Edward Jacobs, Julio Arroyo, Sam Salemizadeh Parizi, Wei Guo, Yong Lu, Rafael Davalos

**Affiliations:** ^1^ Wallace H. Coulter Department of Biomedical Engineering, Georgia Tech and Emory Medical School, Atlanta, GA, USA.; ^2^ Department of Medicine, Houston Methodist and Weill Cornell Medical College, Houston, TX, USA.

## Abstract

Irreversible electroporation (IRE) is a nonthermal ablation modality used clinically for treating unresectable tumors while preserving vital structures through controlled application of pulsed electric fields. Previous data suggest that patient outcomes are enhanced with the induction of an anti-tumor immune response, but current research focuses on using immune checkpoint inhibitors, which function through conventional immune pathways that may be down-regulated by cancer or dysregulated by chemo-induced lymphodepletion. Chimeric antigen receptor (CAR) T cells overcome this limitation, as they are engineered with synthetic receptors that redirect lymphocytes to recognize and target cells expressing tumor-specific structures. CARs are engineered to have an increased binding affinity compared to in situ T-cell binding, amplify internal stimulation cascades, and release pro-inflammatory cytokines that can modulate the endogenous immune system. However, there are still major limitations for adoptive cell therapies in solid tumors, including life-threatening on-target off-tumor cytotoxicity, antigen escape, and failure to infiltrate and persist in solid tumors. Given the substantial evidence that IRE overcomes many of the challenges associated with immune infiltration and persistence in solid tumors, there is a strong premise for using targeted cell therapies following IRE, which would then target residual cancer that could repopulate the lesion. Here, we present the first proof-of-concept combination of IRE with CAR T cells. We validated that the cell membrane CAR target is not affected in electroporated cells that survive IRE, allowing for subsequent binding and elimination of residual tumor. The research demonstrates the feasibility and synergy of a novel combination of 2 clinically used techniques.

## Introduction

Pancreatic cancer is the seventh leading cancer-related death worldwide, with a 5-year survival rate of ~13% [1]. Despite decades of work improving surgical procedures, chemoradiation, and early diagnostic techniques, pancreatic cancer is still an insidious prognosis due to its surreptitious and aggressive growth. Consequently, most pancreatic cancer patients are diagnosed at locally advanced or metastatic stages, with 80% to 90% having pancreatic ductal adenocarcinoma (PDAC) [2]. PDAC is characterized by substantial vascular and ductal involvement that precludes surgical resection in >80% of patients [3]. Further, even for amenable patients who undergo surgical resection, >50% experience local tumor recurrence within a year [4]. The poor tumor location for pancreatic cancer is paralleled by extensive genetic heterogeneity, an impenetrable stroma, and an immunosuppressive “cold” tumor microenvironment (TME), which limits the localization and persistence of targeted therapies [5].

Irreversible electroporation (IRE) is a nonthermal focal pulsed field ablation technique used clinically for the treatment of unresectable and aggressive tumors in the prostate [6–8], kidney [9–11], liver [12], and pancreas [13,14]. Clinical NanoKnife IRE employs 90 pulses of high-voltage (1,000 to 3,500 V) 90-μs monophasic pulses, applied through thin surgical probes placed within or adjacent to the tumor [15,16]. The electric field generated within the tissue from the applied voltage permeabilizes the cellular membrane through the formation of nanoscale pores (electroporation) [17–19]. If persistent, cells die due to inability to maintain homeostasis [20]. Due to nonthermal mechanisms, IRE can ablate large volumes of tissue (>50 cm^3^) without significantly heating the surrounding tissue or structures [21,22], allowing it to be delivered near the bowel [23], ducts [24], mature blood vessels [25,26], and nerves [27,28]. Thus, IRE is one of the few treatments available when current surgical resection and thermal ablation methods are contraindicated. Consequently, IRE nearly triples the median survival of patients diagnosed with stage III locally advanced pancreatic cancer (LAPC) from 6–13 to 17–44 months [29–38].

Although treatment success is not predicated by the induction of a pro-inflammatory response, immune activation correlates with survival outcomes [30]. Both the innate and adaptive immune systems are demonstrated to activate and localize within the TME following IRE [39]. However, systemic anti-tumor responses following IRE are not consistent between patients, which may lead to eventual tumor recurrence. Many patients receive chemotherapy before and/or after IRE treatment [34,36,40], which can induce lymphodepletion and weaken the adaptive T-cell response [41]. In an attempt to more consistently generate a persistent peripheral anti-tumor immune response, IRE has predominantly been combined with immunotherapies (i.e., anti-CTLA-4, anti-PD-L1, and anti-PD-1) [42–45]. He et al. [45] presented promising clinical results when combining IRE with anti-PD-1 in LAPC, achieving an overall survival of 44.3 months compared to the 23.4 months for IRE alone. However, major histocompatibility complex I (MHC I) is significantly down-regulated in many cancer types, precluding the binding of primed T cells, with PDAC patients specifically exhibiting suppression rates of 40% to 100% [46,47]. Without receptors for immune cell recognition, micrometastases or portions of the tumor not directly involved within the ablation [48] are hidden from the heightened immune response and eventually repopulate local and distant sites.

Chimeric antigen receptor (CAR) T cells overcome this limitation, as they are engineered with synthetic receptors that redirect T lymphocytes to recognize and target cells expressing disease-specific surface structures, proteins, and glycans, while the T-cell receptor (TCR)–MHC complex targets processed intracellular proteomes [49–51]. CARs are engineered to have an increase in binding affinity over in situ TCR–MHC binding, and generation II to IV CARs have internal signal cascade amplification, which can lead to higher functionality over conventional TCR pathway activation [52,53]. Further, activated CAR T cells release pro-inflammatory cytokines that modulate the endogenous immune system [49,54,55], sometimes with side effects when excessive [55]. Although CAR-T therapy has achieved unprecedented success in hematopoietic cancers [56], there are still major limitations that must be addressed, including life-threatening on-target off-tumor cytotoxicity, antigen escape, and failure to infiltrate and persist in solid tumors. There are also well-defined pathways that inhibit CAR T-cell immunity and recruitment within the TME, including anti-inflammatory cytokines and chemokines released by immunosuppressive immune cells, physical barriers created by tumor-recruited fibroblasts, and immune inhibitors [55].

Given the substantial evidence that IRE overcomes many of the challenges associated with endogenous T-cell infiltration and persistence in solid tumors that are shared with CAR-T therapy, there is a strong premise for using targeted adoptive cell therapies following IRE, which we hypothesize would then target residual cancer that could repopulate the lesion. Here, we demonstrate a proof-of-concept combination of IRE with human CAR T-cell therapy. We demonstrate that the cell membrane CAR target is not affected in electroporated cells that survive IRE, allowing for subsequent binding and elimination of residual tumor. The research presented demonstrates the scientific and clinical feasibility of the novel combination of 2 actively used clinical techniques.

## Results

### Local tumor recurrence is indicated following IRE of pancreatic cancer in an immunodeficient mouse model

While overall survival and progression-free survival for LAPC have been significantly extended with IRE, curative outcomes are still limited by local and distant recurrence [57–59]. Both IRE and CAR T-cell therapy are immunogenic, with the immune system influencing the response to primary and metastatic disease. Tumor response following IRE is enhanced in immunocompetent mice compared to that in immunodeficient nude mice [58], and recent work indicates that IRE produces an abscopal effect in an immunocompetent contralateral Pan02 pancreatic cancer C57BL/6 mouse model, with the size of the secondary tumors remaining stable following treatment of the primary tumor [60]. However, many patients receive chemoradiation that weakens the immune response; therefore, we investigated tumor control following IRE in immunodeficient Pan02-bearing NOD/SCID/IL2gc-KO (NSG) mice. NSG mice lack functional T, B, and natural killer (NK) cells, allowing for the assessment of IRE without adaptive immune-mediated cytotoxicity of residual disease. Although cell death is indicated <24 h after IRE, the necrotic portions of the tumor need to be cleared by the innate immune system. Together with potential scabbing, the presence of small residual tumors cannot be measured with calipers, so we transfected the Pan02 cells to express firefly luciferase (FLuc) for In Vivo Imaging System (IVIS) use to assess the treatment response via tumor luminescence.

**Fig. 1.**
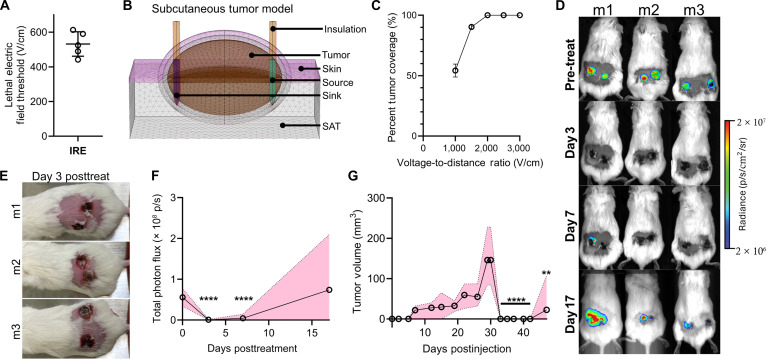
(A) Calculated irreversible electroporation (IRE) lethal electric field thresholds for Pan02 mouse pancreatic cancer using a 3-dimensional (3D) collagen hydrogel; mean ± SD; *n* = 5. (B) COMSOL Multiphysics finite element model (FEM) representing subcutaneous mouse tumors with a 5-mm spacing shown. (C) Simulated percent tumor coverage by the lethal electric field using the subcutaneous FEM with randomized tissue conductivities and tumor sizes (4 to 8 mm); mean ± SD; *n* = 30. (D) In Vivo Imaging System (IVIS) imaging of FLuc-eGFP^+^ Pan02 cells in immunodeficient NOD/SCID/IL2gc-KO (NSG) mice (m) follows complete ablation and subsequent microtumor recurrence after IRE treatment at 2,500 V/cm; *n* = 6. (E) Tumor scabbing and flattening at day 3 post-treatment. (F) Total photon flux within the tumor region of interest from the IVIS images; mean and range presented; multiple 2-tailed *t* tests between the data compared to the initial day 0 total photon flux; *n* = 6. (G) Measured tumor size over time from tumor inoculation; mean and range presented; multiple 2-tailed *t* tests between the data compared to pre-treatment tumor size on day 30; *n* = 6. **P < 0.01; ****P < 0.0001. FLuc-eGFP, firefly-luciferase-enhanced green fluorescent protein. SAT, subcutaneous fat.

To determine the distance-normalized voltage for mouse tumor treatments, we first utilized our Pan02-laden collagen hydrogels to quantify the lethal threshold of the Pan02 cells to conventional NanoKnife IRE (Fig. 1A, 525 ± 77 [442 to 613] V/cm). To account for potential variations in tumor conductivity and size, we simulated the electric field with randomized conductivities and tumor diameters ranging from 4 to 8 mm (Fig. 1B), as previously detailed [61,62]. The percent tumor coverage by the lethal threshold was then calculated in each simulation for applied voltage-to-distance ratios ranging from 1,000 to 3,000 V/cm. From the pre-treatment model, we determined that a voltage-to-distance ratio of 2,500 V/cm should fully ablate every tumor (Fig. 1C). Informed by the computational model, each tumor was then treated, with adjustments to the electrode spacing and voltage made based on the measured tumor size at the time of treatment. A significant drop in luminescence was observed on IVIS imaging on days 3 and 7 (Fig. 1D), indicating complete coverage of the tumor by the ablative field. All tumors were also visibly flattened and scabbed by day 3 post-treatment (Fig. 1E), confirming tumor ablation and justifying the need for imaging to determine tumor response following pulsed electric field treatments. Despite tumor clearance indicated by imaging on days 3 and 7 post-treatment, scabbing was mostly healed, and tumor regrowth was evident in all mice and 66% (4/6) of tumors on day 17. The total measured photon flux within the tumor region of interest (Fig. 1F), as determined from the IVIS images, and the tumor volume over time confirmed partial tumor regrowth in several mice following initial regression (Fig. 1G). Further, for all recurrent tumors, the growths appear to originate in a small region on the edge of the tumor. These findings suggest that while IRE induces acute cytoreduction in pancreatic tumors, residual viable cells can persist and repopulate the lesion in immunodeficient mice. These results underscore the critical role of immune surveillance in achieving durable treatment responses and motivate the development of adjunct immunotherapeutic strategies, such as CAR T-cell therapy, to eliminate post-IRE residual disease.

### CAR target binding is not affected in viable cells following IRE

**Fig. 2.**
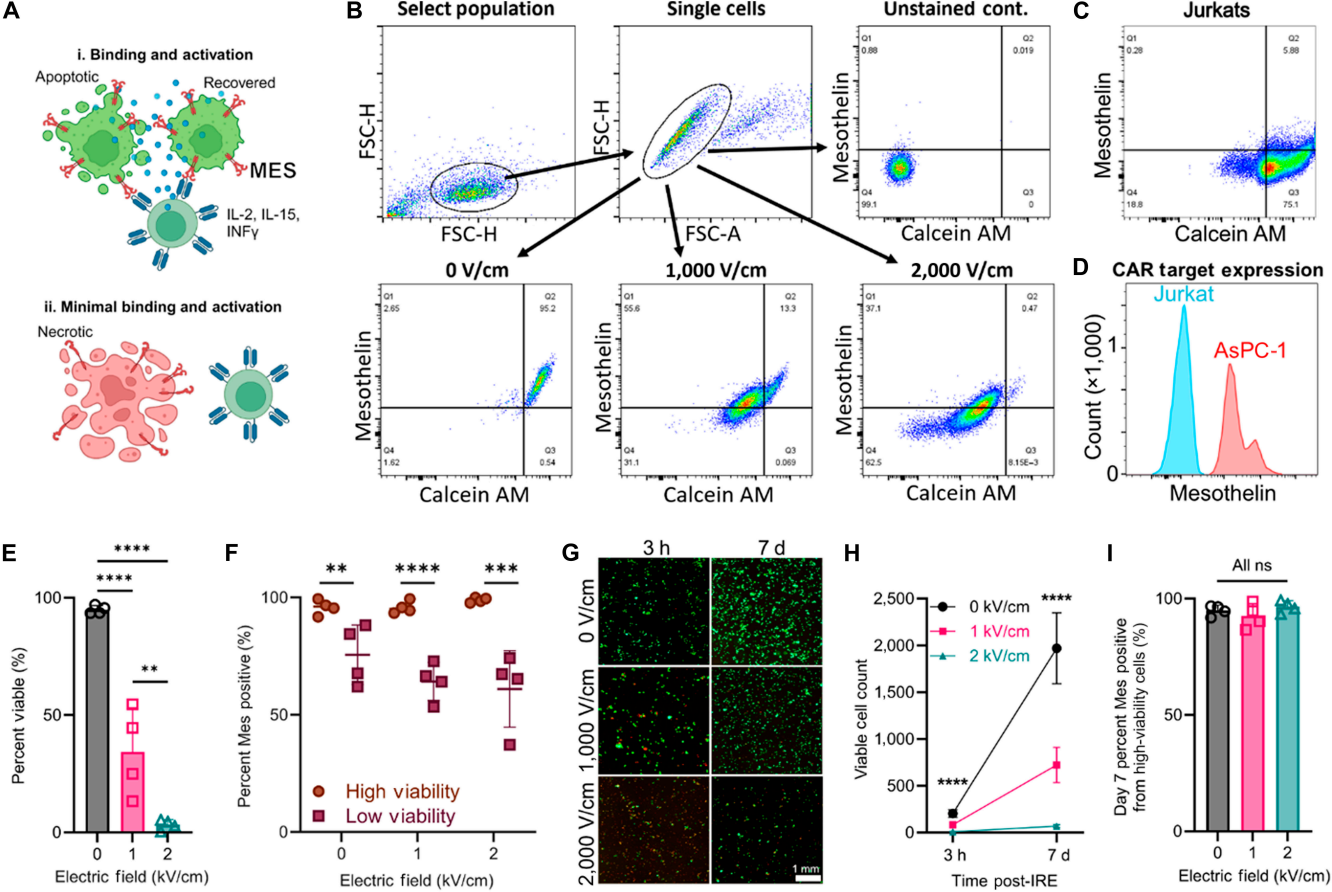
Chimeric antigen receptor (CAR) target binding analysis following irreversible electroporation. (A) Viable and intact cells following electroporation are still available for cell membrane mesothelin (MSLN) binding, while necrotic cells experience a decrease in binding. (B) Flow cytometry gating to isolate single cells and plots of calcein AM versus mesothelin at 3 h following IRE delivery using 0 V/cm (control), 1,000 V/cm, and 2,000 V/cm. (C) Control using mesothelin-negative Jurkats. (D) Mesothelin expression in AsPC-1 cells compared to that in Jurkats. (E) Cell viability 3 h after electroporation at different electric field strengths; one-way analysis of variance (ANOVA) with Tukey’s posttest and correction; mean ± SD; *n* = 4. (F) Percent mesothelin (Mes) expression of high-viability and low-viability cell populations 3 h after electroporation; multiple 2-tailed *t* test; mean ± SD; *n* = 4. (G) Live (green) and dead (red) imaging at 3 h and 7 d after treatment; the scale bar is 1 mm. (H) Viable cell count at different electric fields after IRE and following recovery; one-way ANOVA with Tukey’s posttest and correction within each timepoint; mean ± SD; *n* = 4. (I) Mesothelin binding for recovered cells at day 7; one-way ANOVA with Tukey’s posttest and correction within each timepoint; mean ± SD; *n* = 4. ns, not significant; **P < 0.01; ***P < 0.001; ****P < 0.0001. IL-2, interleukin-2; IL-15, interleukin-15; IFNγ, interferon-γ; FSC-H, forward scatter height; FSC-A, forward scatter area.

Due to the thinness of the cell membrane, the induction of a transmembrane potential during IRE pulse delivery can generate electric fields within the membrane on the order of ~200 MV/m, large enough to potentially destabilize or denature membrane proteins [17,63,64]. Further, the organization and function of membrane proteins are also dependent on lipid interactions, and electroporation physically alters the cell membrane composition and structure [65–67], possibly disrupting the recognizable binding motif within the lipoprotein complex. Therefore, to interrogate whether electroporation precludes the binding of CAR T cells due to dysregulating membrane proteins, we evaluated mesothelin binding via flow cytometry following treatment. Mesothelin is a membrane protein that is commonly overexpressed in human pancreatic cancers and is consequently a frequent CAR target [68,69]. We utilized the AsPC-1 human pancreatic cancer cell line, which is indicated to overexpress mesothelin and has been used for human CAR T-cell experiments [70,71]. Although IRE disrupts cellular homeostasis, residual cellular function and proteins can be partially preserved during delayed cell death. Therefore, the cells were also stained to identify viable cells, with the gating set based on the control, nontreated AsPC-1 cell population (Fig. 2B), to distinguish between high-viability and low-viability populations and between high mesothelin and low mesothelin expression. Compared to Jurkat human leukemia cells, which are negative for mesothelin (Fig. 2C) [72], the wild-type AsPC-1 cells were validated for high mesothelin expression (Fig. 2D).

We next treated AsPC-1 cell suspensions with conventional IRE pulses at different electric fields to induce significantly different brackets of percent viability (Fig. 2E), with no death in the negative control (94.99% ± 1.74% [93.53% to 97.10%]), moderate cell death at 1 kV/cm (34.40% ± 18.65% [13.37% to 54.59%]), and almost complete cell death at 2 kV/cm (3.30% ± 2.19% [0.47% to 5.40%]). The percent high mesothelin expression was then determined within the high-viability and low-viability populations (Fig. 2F). For each treatment magnitude group, the high-viability population had a significantly higher mesothelin expression. However, when examining either the high-viability or low-viability populations, no significant differences in mesothelin expression were observed between the treatments. Subsets of the treated cells were then plated to allow for the viable cells to rebound over 7 d (Fig. 2G). The viability qualitatively matched the trends observed for the percent viability of cells observed with flow cytometry at 3 h post-treatment. For all 3 groups, a portion of the treated cells rebounded (Fig. 2H). Analyzing the viable cells indicated high mesothelin expression in the high-viability population that rebounded following treatment (Fig. 2I), with no significant difference between treatment groups within the high-viability populations at 3 h or 7 d after IRE. These data suggest that residual cancer cells surviving IRE treatment present proteins that CARs can recognize.

### An in vitro assay for monitoring treatment response and tumor recurrence following IRE

**Fig. 3.**
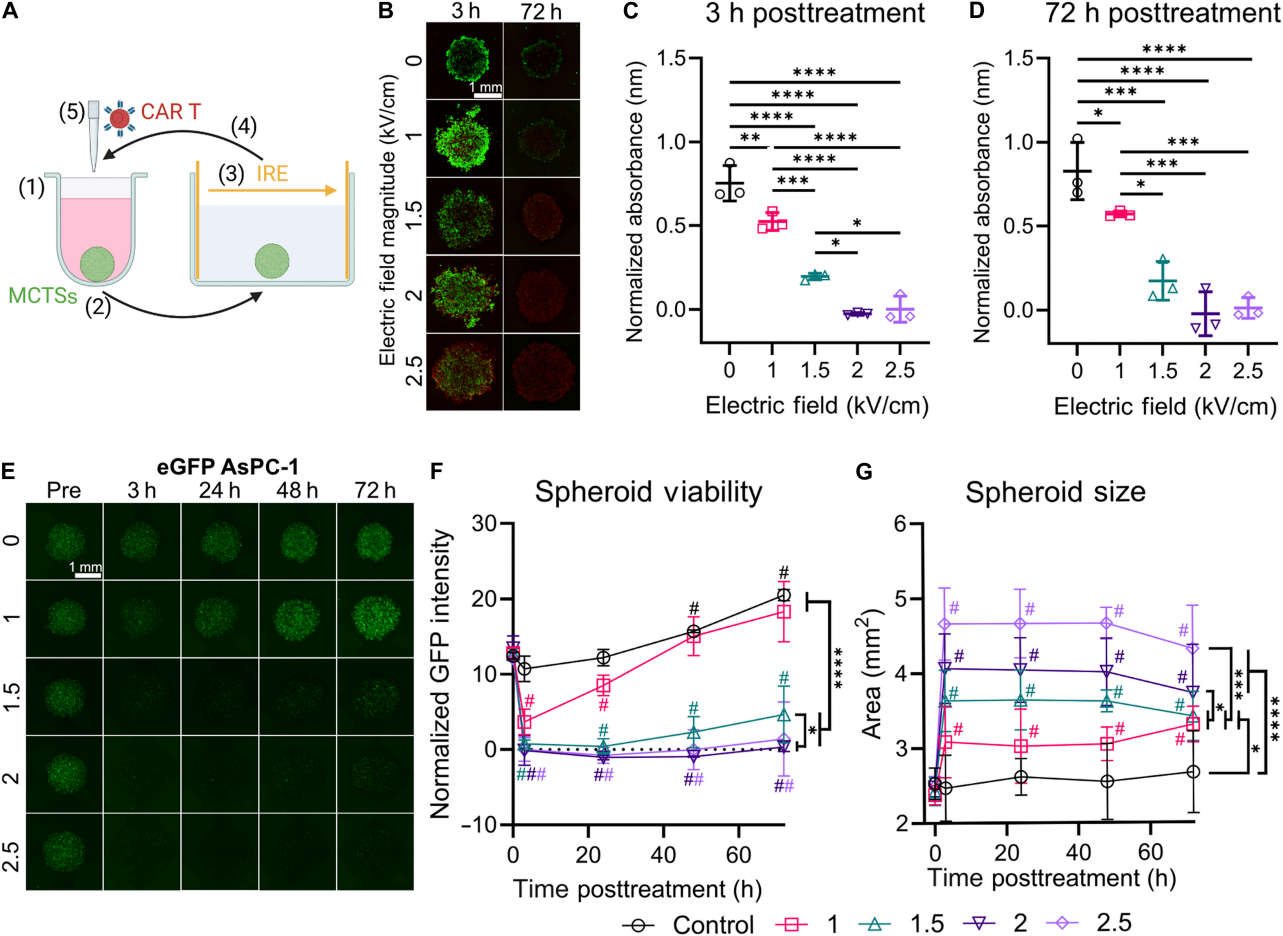
In vitro assay for longitudinal combinatorial treatment evaluation. (A) In vitro multicellular tumor spheroid (MCTS) assay to assess the treatment response to electroporation and CAR T-cell therapy. (1) MCTSs were formed within a low-adherent U-bottom 96-well plate and (2) then moved to a 4-well rectangular plate with low-conductivity buffer to (3) deliver electroporation via parallel-plate electrodes. (4) The MCTSs were immediately moved back into the original U-bottom well, where (5) adjuvant CAR T-cell therapy or sham was delivered. (B) Live (green) and dead (red) imaging of AsPC-1 MCTSs at 3 and 72 h after treatment at different electric field magnitudes; the scale bar is 1 mm. Normalized absorbance for the XTT (sodium 3′-[1-(phenylaminocarbonyl)-3,4-tetrazolium]-bis(4-methoxy-6-nitro) benzene sulfonic acid hydrate) assay at (C) 3 and (D) 72 h post-treatment at different electric fields; one-way ANOVA with Tukey’s post hoc and correction; mean ± SD; *n* = 3. (E) Green fluorescent intensity of FLuc-eGFP^+^ AsPC-1 MCTSs over time and across different electric field intensities. (F) Normalized green fluorescent protein (GFP) intensity and (G) MCTS area over time; one-way ANOVAs with Tukey’s post hoc between groups on the last timepoints (*P < 0.05; ** *P* < 0.01; *** *P* < 0.001; **** *P* < 0.0001); multiple one-sample Wilcoxon signed-ranked tests between that timepoint and the initial zero timepoint (^#^*P* < 0.05); *n* = 3.

While the protein targets for CAR binding are still functional in viable cells following electroporation, it is necessary to validate that the target protein is recognizable by CAR T cells for subsequent removal. Here, we employed a tumor spheroid viability assay as previously described for high-throughput CAR T-cell analysis [73], with modifications to include IRE treatments within the pipeline (Fig. 3A). In vitro tumor spheroids provide a bridge between computational modeling and in vivo validation to determine treatment efficiency and explore biological mechanisms in a reproducible and high-throughput system [74,75]. Three-dimensional (3D) cultures have emerged to achieve higher biomimicry of cell–cell and cell–extracellular matrix interactions [76,77], gene expression, cellular heterogeneity, and microarchitecture of the native tissue [78–80]. Consequently, 3D models improve the predictability of therapeutic toxicity and sensitivity, with significantly higher drug resistance observed in 3D models than in 2-dimensional models [81]. Given their unique advantages, developing in vitro 3D models has been of growing interest within adoptive cell therapy development for assessing target binding affinity, comparing treatment efficacies, and determining patient-specific treatment responses before clinical delivery [82].

Multicellular tumor spheroids (MCTSs) were fabricated using mesothelin-positive (MSLN^+^) AsPC-1 cells, and an initial study was performed to evaluate the treatment response to IRE at different electric field strengths (1 to 2.5 kV/cm). Similar to the suspension and flow cytometry data, the live/dead staining revealed a stark decrease in viability for all treatment groups at 3 h post-treatment that persisted at 72 h post-treatment (Fig. 3B). To validate the observed trends in viability, we utilized a quantitative XTT (sodium 3′-[1-(phenylaminocarbonyl)-3,4-tetrazolium]-bis(4-methoxy-6-nitro) benzene sulfonic acid hydrate) assay. The tetrazolium salt is reduced to a formazan product, which is directly proportional to the number of viable and metabolically active cells. There was a significant decrease in metabolic activity for all treatment groups (1 to 2.5 V/cm) when compared to that of the control, at both 3 and 72 h post-treatment (Fig. 3C and D). The metabolic activity of MCTSs treated with 1 and 1.5 V/cm was significantly different from that of every other group at 3 h, but the metabolic activity for the 1.5 V/cm treatment significantly decreased by 72 h, with no significant differences to the 2.0 and 2.5 V/cm groups. These data support that cell death and recovery following IRE are dynamic, providing a rationale for the need to track treatment response at multiple timepoints. However, the XTT assay has limitations for tracking spheroid viability with cellular therapies, as the measured metabolic activity will be influenced by the activation and proliferation of CAR T cells, especially with concurrent cell death following IRE.

To enable longitudinal and isolated tracking of MCTS response with CAR T-cell treatments, we transfected MSLN^+^ AsPC-1 cells with a firefly-luciferase-enhanced green fluorescent protein (FLuc-eGFP) lentivirus (Fig. 3E). We observed a stark decrease in eGFP fluorescent intensity as soon as 3 h following IRE for all treatment groups compared to the control, and we continued to measure the average green fluorescent protein (GFP) intensity within the MCTSs to quantify viability for up to 72 h (Fig. 3F). The GFP intensity at 3 h dropped significantly from baseline for all treatment groups. However, for 1 and 1.5 kV/cm, the GFP intensity then increased over the following 69 h, with significantly higher GFP intensities at 72 h compared to those at 3 h, indicating incomplete treatment and proliferation of surviving cells within the tumor spheroid. The 2 and 2.5 kV/cm groups did not significantly change from their initial drop at 3 h. These data validated the viability data observed from the live/dead imaging and metabolic assays, providing a novel method for tracking individual tumor spheroid viability and proliferation for longitudinal electroporation studies.

Following treatment, the size of the MCTSs significantly increased for every IRE group compared to their pre-treatment measurements (Fig. 3G). The spheroid size did not change significantly for the control across all timepoints and remained unchanged for all IRE groups after the initial significant increase in size measured at 3 h. Further, the change in spheroid size increased significantly with higher applied electric fields. Since the metabolic activity decreased with increased electric field and the initial number of cells within the spheroid was equal across all replicates, the increase in MCTS size cannot be attributed to proliferation but is due to electroporation dysregulating the MCTS organization.

### In vitro spheroid study supports that CAR T-cell therapy can eliminate residual cancer following IRE

**Fig. 4.**
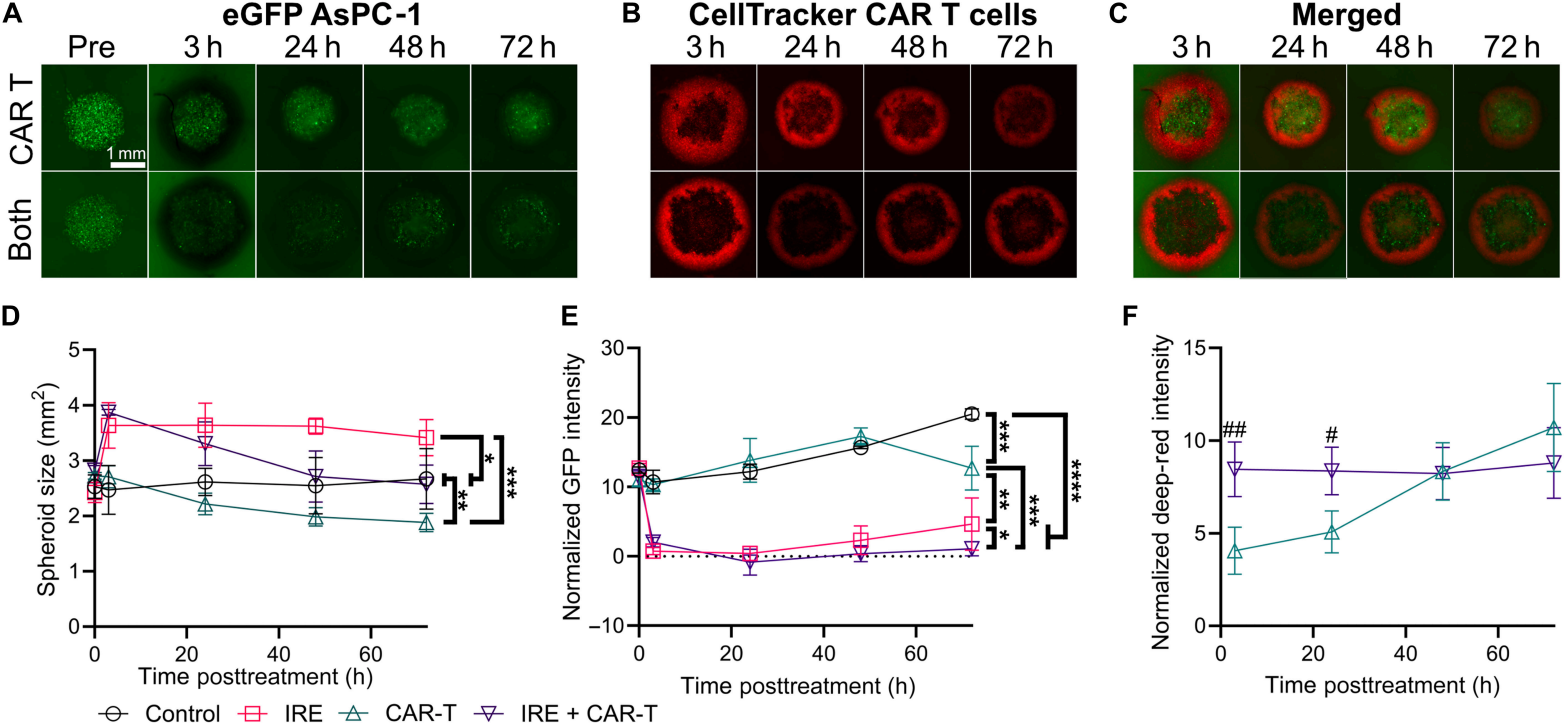
In vitro evaluation of anti-tumor efficacy and infiltration following IRE and CAR T-cell therapy. (A) Green fluorescent intensity of FLuc-eGFP^+^ AsPC-1 MCTSs, (B) deep-red intensity of CellTracker-stained CAR T cells, and (C) merged images over time for the CAR-T-cell-only and combinatorial treatments (both). (D) Measured MCTS area and (E) normalized eGFP intensity over time; one-way ANOVAs with Tukey’s post hoc between groups on the last timepoints (*P < 0.05; **P < 0.01; ***P < 0.001; ****P < 0.0001); *n* ≥ 3. (F) Comparison of deep-red intensity within the tumor spheroid over time; 2-tailed *t* tests between groups at each timepoint (^#^*P* < 0.05; ^##^*P* < 0.01); *n* ≥ 3.

Utilizing the MCTS assay, we observed that the cellular organization was disrupted following IRE, and proliferation was preserved in portions of the MCTSs for applied electric fields below 1,500 V/cm. To investigate whether CAR T-cell therapy can eliminate residual cancer and whether the lower density facilitates CAR T-cell penetration into the MCTSs, we utilized our FLuc-eGFP^+^ AsPC-1 MCTS assay again, with the addition of adjuvant CAR T cells (Fig. 4A to C). CAR T cells were fabricated using primary human T cells as previously described [83–85] to express a second-generation anti(α)-human(hu)MSLN-m28-mCD3z CAR gene against huMSLN [86–88]. CAR T cells were added to spheroids at a 1:5 ratio of tumor cells to T cells. The size of the spheroids treated with only CAR T cells decreased significantly over time, in contrast to that of the controls, which remained steady (Fig. 4D). The size of the spheroid again increased significantly at 3 h following 1,500 V/cm IRE due to dysregulation of the MCTSs. However, with the subsequent addition of CAR T cells, the size then decreased significantly over time, suggesting a potential combinatorial effect on the cells within the MCTSs. The improved cytotoxicity from the combinatorial therapy was further supported by the normalized GFP intensity within the spheroid (Fig. 4E). The GFP intensity within the combinatorial treatment dropped by 3 h and remained low, with a significantly lower GFP intensity than those of the other groups, including the IRE-only group that showed evidence of partial recurrence by 72 h. Although the size of the MCTSs decreased over time within the CAR T-cell group, the fluorescent intensity was not significantly different from that of the control until 72 h, when the GFP intensity decreased significantly. This suggests that the CAR T cells are binding and inducing cytotoxic effects on the outer layers of the MCTSs without deeper penetration until 72 h. To investigate CAR T-cell localization within or around the MCTSs, we stained CAR T cells with CellTracker Deep Red. Using the eGFP images, we defined a region of interest outlining the MCTS border and then overlaid it on the CellTracker Deep Red images to quantify the normalized deep-red intensity within the MCTSs (Fig. 4E). The deep-red intensity was initially significantly higher within the MCTSs following IRE. Measuring over 72 h, the intensity remained unchanged within the combinatorial group but significantly increased within the CAR-T-cell-only group (Fig. 4F). The increase in infiltration by 72 h within the CAR-T-cell-only group corresponds with the decrease in spheroid size and decrease in viability at 72 h, verifying the successful generation of anti-tumor CAR T cells and validating cytotoxicity against cancer cells expressing bindable CAR targets. The initially higher infiltration within the combinatorial group suggests that the disrupted MCTS allows for the penetration of CAR T cells; however, due to the significantly lower viability and subsequent lower presence of bindable CAR targets, the CAR T cells do not have pressure to invade. Further, the data indicated that there is an increase in the efficacy of combinatorial IRE and CAR T-cell therapy, preventing the few viable cells from repopulating the MCTSs and that many of the CAR T cells within the combinatorial group did not need to invade for additional cytotoxic effects.

### Combinatorial IRE and CAR T-cell therapy enhance efficacy in a preclinical human pancreatic cancer mouse model

**Fig. 5.**
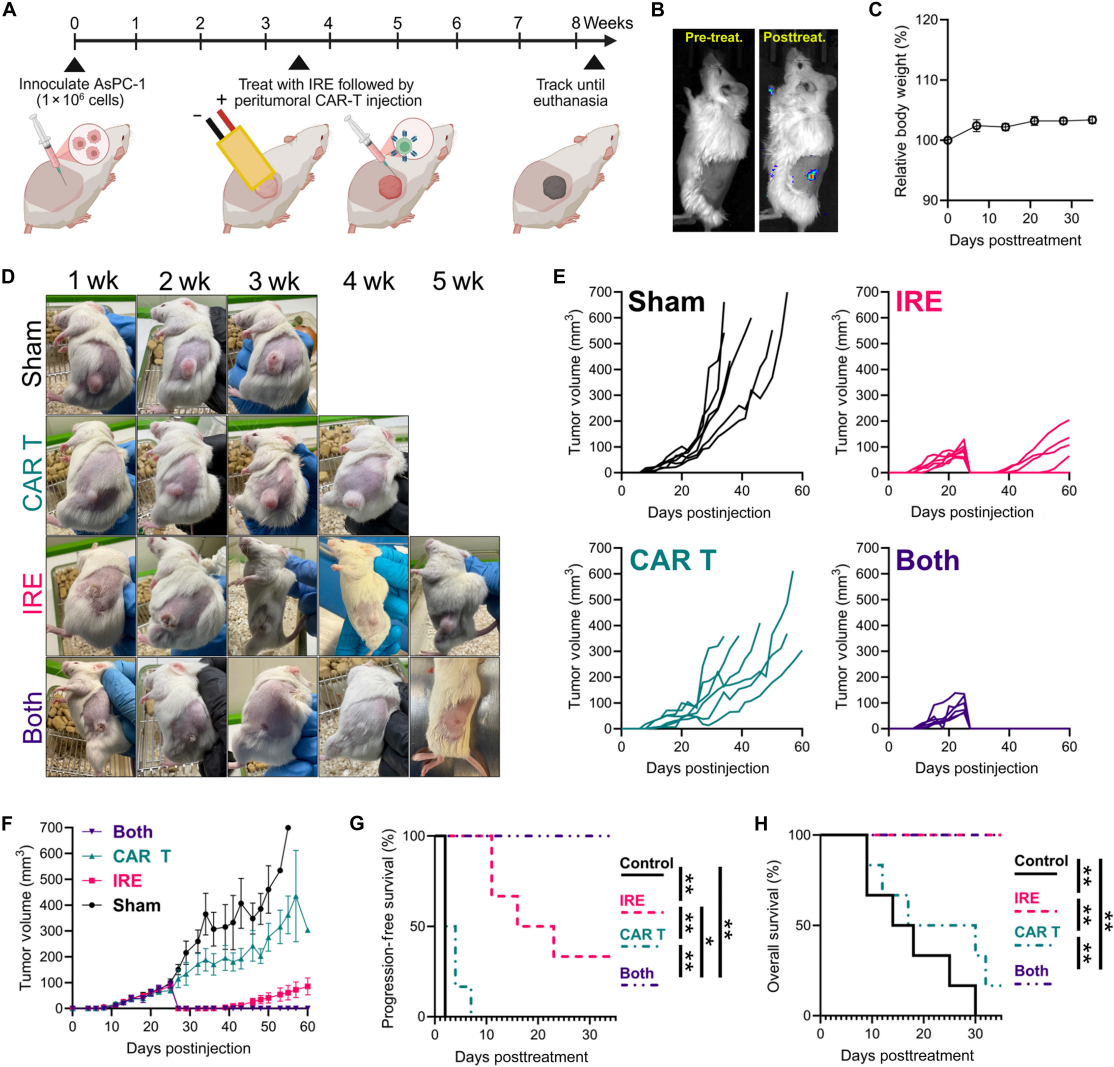
Subcutaneous mouse model of human pancreatic cancer for comparing tumor response and survival following combinatorial IRE and CAR T-cell treatment. (A) Schematic of the treatment timeline: NSG mice were inoculated with MSLN^+^ AsPC-1 cells on day 0. Mice received IRE followed by peritumoral CAR T-cell injection on day 25 and were tracked for 35 d until day 60. (B) Pre- and post-treatment IVIS imaging verifies successful peritumoral FLuc^+^ αMLSN CAR T-cell injection. (C) Relative rodent weight over time post-treatment; mean ± standard error of the mean (SEM); *n* = 24. (D) Representative images of rodents over the post-treatment tracking period (blanks for mice sacrificed). (E) Tumor measurements from inoculation for all mice. (F) The average measured tumor volume from inoculation; mean ± SEM; *n* = 6 (the sample size drops as mice reach the tumor size endpoint). (G) Progression-free survival and (H) overall survival for each group; Kaplan–Meier with Bonferroni multiple comparisons; *n* = 6 (*P < 0.05; **P < 0.01).

While in vitro studies are essential for evaluating novel CAR designs, the limitations for CAR T cells are well-documented in vivo and cannot be fully recapitulated in vitro [55,69,82]. Immunotherapies, including CAR T-cell therapies, are also required to be evaluated in vivo for efficacy and are shown to be mouse strain dependent, highlighting the need for appropriate strain selection for specific studies [89–91]. Severely immunodeficient NSG mice are the most common model in adoptive cell therapy research, as they can engraft human cancers, enabling the development of patient-specific therapies [89]. To validate the proof-of-concept combination of IRE and CAR T-cell therapy, as demonstrated by the in vitro MCTS assay, we performed a 60-d survival study (Fig. 5A) in which mice were tracked for 5 weeks after treatment. NSG mice were inoculated with 1 × 10^6^ nontransfected MSLN^WT^ AsPC-1 cells. The tumors were treated with IRE as detailed above, with an immediate local peritumoral injection of 5 × 10^6^ αhuMSLN CAR T cells. To visualize CAR T-cell localization and verify successful transfection, the CAR gene was concomitantly expressed with firefly luciferase. IVIS imaging, both before and after treatment, verified the successful delivery of CAR T cells locally (Fig. 5B). All mice showed a steady, continuous increase in body weight, in line with that of healthy mice of their age and sex (Fig. 5C). Further, wellness checks by the researchers and the department of animal research staff did not indicate cytotoxicity associated with treatment, with early euthanasia all performed due to tumor sizes growing to 12 mm in any direction (Fig. 5D and E). These data and observations suggest that the combinatorial treatment was well tolerated. For both the combinatorial and IRE-only groups, tumors were eradicated within 48 h of treatment, whereas none of the control or CAR T cell groups disappeared. Both the combinatorial and IRE-only groups resulted in tumor scabbing similar to that in the in vivo Pan02 IRE treatments, which resolved by ~14 to 21 d post-treatment (Fig. 5D). A small amount of pigmentation from scarring persisted after the scabbing healed in the IRE-only and combinatorial groups. Similar to the in vivo Pan02 IRE treatments, a few (4/6) of the IRE tumors recurred during follow-up (Fig. 5D and E). However, none of the combinatorial groups had tumor recurrence during the 5-week follow-up. Both the IRE-only and combinatorial groups significantly increased the progression-free and overall survival, compared to the sham and CAR-T-only groups. The combination of IRE and CAR T-cell therapy significantly increased the progression-free survival (Fig. 5G) but not the overall survival (Fig. 5H) over that of the IRE-only group within the 5-week post-treatment follow-up. These data validate the in vitro results and support the improved therapeutic efficacy of combinatorial IRE and CAR T-cell therapy.

## Discussion

Here, we demonstrate the scientific and clinical feasibility of combining IRE with engineered cells and support the use of pulsed field ablation techniques with an adoptive cell therapy, specifically CAR T-cell therapy. Our findings suggest that IRE does not compromise the structural integrity of membrane-bound tumor antigens in cells that survive treatment, thereby preserving the binding potential of CARs. This mechanistic insight is critical, as other ablation techniques use thermal energy, which can indiscriminately denature proteins, potentially limiting compatibility with targeted immunotherapies. In contrast, IRE uniquely retains epitope integrity in viable cells within the treatment margin, enabling CAR T cells to recognize and eliminate residual disease. This highlights the potential for IRE to serve not only as a cytoreductive tool for otherwise unresectable tumors but also as a unique facilitator of antigen-targeted immune engagement in solid tumors commonly treated by thermal ablation.

Nevertheless, mesothelin is a cell surface glycoprotein with a heavy and light chain that may not denature as easily from high electric fields as other CAR targets that are complexes composed of many constitutive parts (e.g., HER2 and IL13Rα2). Thus, evaluations similar to those used to investigate mesothelin binding through flow cytometry should be performed to verify the recognition of other CAR targets following electroporation. In addition to direct disruption, there are many theorized mechanisms of cell death following IRE that can occur concomitantly and evolve over hours to days [92]. Programmed cell death mechanisms, such as autophagy [93], may also be involved in this process or exert a bystander effect on nearby tumor cells, which could indirectly affect membrane protein expression. High-voltage pulsed electric fields also affect the cellular cytoskeleton, which is structurally and functionally linked to the cellular membrane and transmembrane proteins. This can potentially affect tumor antigen binding and cell–cell adhesion that influences immune infiltration. While adjuvant therapies would not be delivered after tumor recurrence, future work should also analyze recurrent tumors following IRE to validate similar CAR antigen expression in these tumors compared to untreated antigen-positive control tumors.

We contend that IRE and CAR T-cell therapy may also synergize their treatment pipelines. Many patients receive chemotherapy before IRE [34,36,40], so T cells can be removed to manufacture CAR T cells ex vivo for reinfusion at treatment. This combination can then overcome the aspects of a weakened anti-tumor immune response due to lymphodepletion and lack of endogenous T-cell priming in these patient populations [41]. Delivery of NK cells and allogenic γδ T cells has been briefly demonstrated concomitant with IRE in humans [94–96], but NK cells do not generate a sustained or specific anti-tumor immune response [97]. Further, while γδ T cells are less restricted by specific tumor antigens, potentially enabling broader cancer recognition, they still require MHC I binding, which may be down-regulated. Aggressive cancers can similarly escape CAR T-cell therapy by down-regulating the target antigen [98]. Tandem CAR T cells aim to overcome this by targeting multiple antigens simultaneously, decreasing the chance that cells can down-regulate both targets. Dual and triple CAR T cells have been demonstrated to increase therapeutic efficacy but have the chance to significantly increase on-target off-tumor cytotoxicity [99]. Cytopenias and neurotoxicity are also directly correlated with CAR T-cell concentration [55], which can limit the efficacy of the therapy, as high concentrations are typically required for solid tumors. Reducing the necessary CAR T-cell concentration with cytoreductive IRE may allow for the safer use of CAR T-cell variations. The AsPC-1 tumors utilized here were wild-type for mesothelin, but future work should investigate if cytoreduced IRE can decrease the concentration of CAR T cells necessary using controlled levels of antigen expression, heterogeneous antigen expression, or mixtures of antigen-positive and antigen-negative cells.

Multiple cell populations contribute to the immunosuppressive TME, including differentiated cancer cells, cancer stem cells, tumor-associated fibroblasts, and immunosuppressive immune cells [5]. IRE acts indiscriminately on proliferating and nonproliferating cells within the lethal electric field [100], producing submillimeter demarcations between ablated and nonablated tissue. Therefore, recalcitrant (e.g., cancer stem cells) and immunosuppressive immune cells are removed in addition to bulk tumor cytoreduction [37,43,101]. IRE also alters the physical properties of the TME by reducing extracellular matrix density and rigidity [42,102], as well as increasing tumor-associated blood vessel permeability [102–105]. Together, this reverses stroma-induced immunosuppression and tumor-associated hypoxia [42]. For all tumors that recurred following IRE in both the Pan02 IRE treatments and AsPC-1 IRE treatments, we observed that regrowth typically occurred at the edge of the scabbing. As the electric field magnitude decreased away from the electrodes, a few cells were potentially not above the lethal electric field. This highlights the need for adjuvant therapeutics that can remove potentially viable cancer that repopulates the lesion.

A previous study found that 5 × 10^6^ MSLN CAR T cells alone could not clear the primary pancreatic tumor in NSG mice but significantly prevented the initiation and progression of lung metastases [106], informing the concentration used for this proof-of-concept combination of IRE with CAR T-cell therapy. We found similar results for the standalone CAR T-cell group, with a nonsignificant decrease in tumor growth and a nonsignificant increase in progression-free and overall survival compared to those of the control. Within this study, we investigated the local peritumoral delivery of CAR T cells after IRE. Local delivery is demonstrated clinically to achieve reduced side effects and higher efficacy than systemic delivery, although with many of the same limitations due to the immunosuppressive TME. As IRE is delivered percutaneously, with electrodes placed within and around the tumor, we contend that local delivery through electrodes would be feasible and clinically preferable to systemic delivery for the control of LAPC. However, pancreatic cancer is often metastatic, so future work should investigate how IRE may improve the infiltration of systemically delivered CAR T cells into the TMEs of IRE-treated primary tumors and nontreated metastatic tumors.

**Fig. 6.**
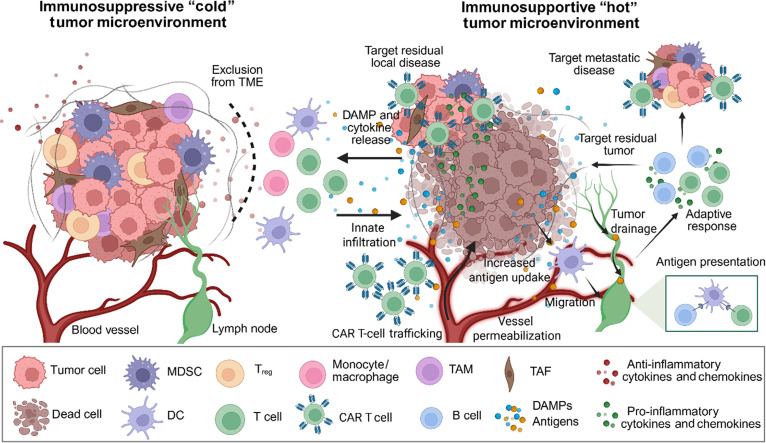
Summary and future directions. IRE indiscriminately ablates cells within the dense and immunosuppressive tumors, clearing the regulatory T cells (T_reg_), tumor-associated macrophages (TAMs), myeloid-derived suppressor cells (MDSCs), and the bulk tumor. Post-ablation, systemic CAR T-cell therapy can then effectively persist within the tumor microenvironment to clear the residual tumor. Adapted from Jacobs et al. [15] (Creative Commons Attribution 4.0 International License). TME, tumor microenvironment; DAMP, damage-associated molecular pattern; DC, dendritic cell; TAF, tumor-associated fibroblast.

The application of either IRE or CAR T cells as a monotherapy has proven successful in enabling an endogenous anti-tumor response through the induction of pro-inflammatory cytokines, facilitating local immune cell recruitment. Additionally, IRE has been shown to activate systemic anti-tumor response, resulting in the reduction of distant metastasis in rodents [103], similar to the “abscopal effect” that is rarely observed in patients exposed to radiotherapy. With the sequential delivery of IRE and CAR T cells, the combinatorial treatment offers the potential to more consistently generate a robust local and systemic immune response that can persist beyond the initial treatment by priming the adaptive immune response. An adaptive immune response may then recognize the cancer that has down-regulated the CAR target. While this work demonstrated a proof of concept for treating human disease, future work should utilize a synergistic tumor and allogenic CAR T-cell combination within an immunocompetent model to investigate the use of IRE for facilitating the infiltration and persistence of adoptive cell therapies in an immunogenically active TME (Fig. 6). We expect that CAR-T infiltration and persistence will be significantly improved following IRE, with increased recruitment of the endogenous immune system. This may then “wake up” a potentially dysregulated immune response to respond to local and metastatic disease (Fig. 6).

## Methods

### Cell culture

Pan02 mouse pancreatic cancer cells (Cytion, 300501), AsPC-1 human pancreatic cancer cells (American Type Culture Collection [ATCC], CRL-1682), and Jurkat immortalized human T lymphocytes (ATCC, TIB-152) were cultured in RPMI 1640 medium (Thermo Fisher, 11875093) supplemented with 10% (v/v) fetal bovine serum (Fisher Scientific, FB12999102) and 1% (v/v) 10,000 U/ml penicillin–streptomycin (Gibco, 16140122). The cells were maintained at 37 °C and 5% CO_2_ in a regulated, humidified incubator (Fisher Scientific, 13-998-086). Pan02 cells and AsPC-1 cells were passaged between 70% and 90% confluency, and Jurkats were maintained in cell suspension between 1 × 10^6^ and 2 × 10^6^ cells/ml. All cells were utilized between passages 10 and 20.

### Cell transfection with reporter FLuc-eGFP

Pan02 or AsPC-1 cells were plated at 1 × 10^4^ per well into a 96-well plate and incubated overnight. A 2-fold titration of a FLuc-eGFP lentivirus (BPS Bioscience, no. 79980), driven under a single cassette by the cytomegalovirus (CMV) promoter for co-expression, was performed from a max multiplicity of infection of 100, with the lentivirus diluted in supplemented cell culture media (as described in the “Cell culture” section). The cells were incubated for 8 h, then the medium–lentivirus mixture was replaced with fresh supplemented cell culture media. The cells were incubated for 3 to 5 d until confluent, with transfection confirmed through green fluorescence from eGFP using a fluorescence microscope (Leica, LMI8). The cells were then fully passaged into a 6-well plate for expansion. Once confluent, the cells were released and suspended in 10 ml of supplemented media for cell sorting using a 9-color FACSMelody cell sorter (BD Biosciences). Following gating for the cells and isolation of single cells, the cells were then gated for high eGFP expression, and the population was collected. The cells were then resuspended for cell culture and further expansion. Stocks of FLuc-eGFP Pan02 and AsPc-1 cells were frozen in liquid nitrogen.

### NSG mice

NSG mice were obtained through breeding at the Georgia Tech Department of Animal Research (GT DAR), with ages ranging from 5 to 7 weeks at the start of experiments. Equal numbers of male and female mice were used. All rodent experiments were conducted at GT DAR under Institutional Animal Care and Use Committee approval (protocol no. A100702) at Georgia Tech and in accordance with the National Institutes of Health (NIH) Guide for the Care and Use of Laboratory Animals. Mice were maintained in sterile housing with food, water, and cage changes occurring under fumigation.

### Pan02-NSG local tumor recurrence experiments

FLuc-eGFP Pan02 cells were washed 2 times with sterile Hank’s balanced salt solution (HBSS; Thermo Fisher, 14025092), resuspended at 20 × 10^6^ cells/ml, and then mixed with an equal volume of sterile Matrigel (Corning Matrigel Matrix, CB-40234C). At GT DAR, NSG mice were subcutaneously inoculated with 100 μl of the cell suspension after the site was shaved and sterilized with an isopropyl wipe. Injection sites were measured 3 times a week to monitor tumor size. Mice were treated once the tumors reached >100 mm^3^, where $\text{volume}=0.5\times {\text{length × width}}^{2}$. Prior to IRE pulse delivery, the mice were initially imaged using IVIS at GT DAR. Briefly, 10 μl of a 15 mg/ml d-luciferin (GoldBio, LUCK-1G) in sterile HBSS was injected intraperitoneally, with serial imaging performed every 2 min for 45 min to determine the optimal imaging time window. Following imaging, IRE was delivered through custom 2-needle electrodes, as previously described [61,62], using a custom pulsed electric field generator (OmniPorator, Vitave Inc., Czech Republic). Conventional 90 bursts of 90-μs pulses were applied at a rate of 1 Hz. The applied voltage was adjusted to deliver 2,500 V/cm of center-to-center electrode spacing, with the spacing set to be ~1 mm less than the max tumor diameter to facilitate insertion into the tumor. The applied voltages and currents were recorded using a WaveSurfer 5-GHz oscilloscope (Teledyne LeCroy, 4024HD) equipped with a 1,000× attenuated high-voltage probe (Siglent, DPB5700) and a 10× attenuated current probe (Pearson Electronics, 3972). Following treatment, the needle insertion sites were inspected for potential bleeding, and the animals were recovered. Mice were then imaged again using IVIS on day 3, day 7, and day 17. In vivo imaging luminescent data were collected and analyzed with Living Image 4.4.5 (PerkinElmer).

### Mesothelin binding and viability following electroporation

AsPC-1 cells (1 × 10^6^) were suspended in osmotically balanced low-conductivity buffer [107], and 100 μl was pipetted into a sterile 4-mm cuvette (BTX, no. 45-0142). Pulsed electric fields were delivered using the custom generator and pulsing scheme described above at 0, 400, and 800 V to generate electric field magnitudes of 0, 1,000, and 2,000 V/cm, respectively. The treated cells were then either transferred to a 24-well plate with fresh cell culture media or to a 1.5-ml tube with 400 μl of cell culture media for flow cytometry. For the plated cells, the cell culture medium was replaced on day 3, and on day 7, they were released to resuspend in media in a 1.5-ml tube for flow cytometry. The processing and flow cytometry steps were identical for both the 3-h and 7-d timepoints, with the timing for the 3-h group adjusted so that flow cytometry occurred at 3 h following treatment. Cell suspensions were centrifuged at 500 × g, with the liquid aspirated and resuspended in 500 μl of flow cytometry buffer (Thermo Fisher, 00-4222-26). The cells were again centrifuged and resuspended with 1 μl (10 μl/10^6^ cells) Human Mesothelin Alexa Fluor 488-conjugated Antibody (R&D Systems, FAB32652G) and 0.5 μl of Invitrogen CellTrace Calcein Red-Orange, AM (Fisher Scientific, 50-113-7411) in 500 μl of flow cytometry buffer. Tubes were placed on a tube rocker on low within a dark 4 °C refrigerator for 45 min. The cells were then washed twice with flow cytometry buffer to remove excessive stain, resuspended in 500 μl of flow cytometry buffer, and then analyzed using Cytek Aurora (Cytek Biosciences). Flow cytometry data were collected with BD FACSDiva v8 (BD Biosciences, San Jose) and analyzed using FlowJo X v11 (FlowJo, Ashland). Gates for viability and mesothelin expression were established using the unstained and stained antigen-positive control (AsPC-1) samples and the antigen-negative control (Jurkat) samples, with guidance from the flow cytometry core. Gates were maintained for each subsequent experiment.

Plated cells not used for flow cytometry were stained for viability at 3 h and 7 d following treatment. The cell culture medium was replaced with a live/dead stain consisting of 0.5 μl of Invitrogen CellTrace Calcein Green, AM (Thermo Fisher, C3100MP) and 2.5 μl of propidium iodide (Thermo Fisher, P3566) in 250 μl of phosphate-buffered saline (Thermo Fisher, 10010023), and then the cells were imaged using a Leica DMI8 fluorescence microscope. Cells were counted within the Leica LasX software within the region of interest corresponding to the image gathered using the ×10 objective.

### CAR T-cell fabrication

Human T cells were isolated from the peripheral blood mononuclear cells of a healthy donor using Human CD3+ T cell Isolation Kit (STEMCELL, no. 17751) and activated using αCD3/CD28 Dynabeads (Thermo Fisher, no. 11132D) and 200 U/ml human interleukin-2 (hIL-2; R&D Systems, no. 202-IL) for 24 h. During the activation, T cells were transduced with hMSLN-(M5)-h28Z-Luc-CAR encoding γ-retrovirus [108] to make T cells targeting human MSLN by centrifugation at 1,800 rpm for 2 h at room temperature. T cells were then expanded in the presence of 100 U/ml hIL-2 and 2-mercaptoethanol (0.1% of the total culture medium volume) for an additional 7 to 8 d before use.

### MCTS experiments

To create MCTSs, either AsPC-1 or FLuc^+^eGFP^+^ AsPC-1 cells were passaged and resuspended in supplemented cell culture media at 5 × 10^5^ cells/ml, with 100 μl pipetted into the wells of a 96-well ultralow-adherent plate [109]. The plate was centrifuged at 150 × g for 5 min, and the cells were monitored for 2 d to verify MCTS formation. To apply IRE, the spheroids were removed and placed in a rectangular 4-well plate (Ibidi) containing 400 μl of low-conductivity buffer. Custom flat-plate electrodes with an 8.5-mm spacing were used to deliver uniform pulsed electric fields, with the voltages and currents monitored as described above. The MCTSs were then placed back into the ultralow-adherent plate with fresh 200 μl of supplemented cell culture media. CAR T cells were stained for 30 min with 1 μM CellTracker Deep Red (Thermo Fisher, C34565). For groups receiving CAR T-cell treatments, CAR T cells were added at a 5:1 ratio to the pre-treatment AsPC-1 cell count (i.e., 25 × 10^5^ cells). The viability of nontransfected AsPC-1 cells was imaged using the live/dead stain detailed above. The XTT assay was performed according to the manufacturer’s instructions; 50 μl of the XTT-activated assay reagent was added to each well and incubated for 18 h. The supernatant within each well was then transferred to a 96-well plate, and absorbance was recorded at 475 and 660 nm (Biotech Synergy HT). The measured absorbance at 475 nm was normalized using the readings at 660 nm. The size and fluorescent intensity of the FLuc^+^eGFP^+^ AsPC-1 MCTSs were quantified within ImageJ (NIH). Each image was separated into the individual channel components (i.e., bright field, green, and deep red). A region of interest, informed by bright-field imaging and green fluorescence, was drawn around the border of the MCTSs. The intensities of both the green and deep-red channels were measured within the MCTSs and outside to obtain background intensity, with the color intensities normalized by subtracting the background intensities.

### AsPC-1 NSG mouse experiments

AsPC-1 cells (1 × 10^6^) were prepared and subcutaneously inoculated into NSG mice as described above for the Pan02-NSG experiment, with the injection sites measured 3 times a week to monitor tumor size. Mice were randomly grouped and treated at day 23 with either (a) sham electrode insertion with local saline injection, (b) IRE with local saline injection, (c) sham electrode insertion with local CAR T-cell injection, or (d) combinatorial treatment using IRE with local injection of CAR T cells within 1 min following treatment. IRE delivery was performed as detailed above. The local injections were performed slowly to prevent backflow, and for CAR T-cell delivery, 5 × 10^6^ cells were injected in 100 μl of sterile saline. The mice were imaged before and after treatment using IVIS. Following treatment, the needle insertion sites were inspected for potential bleeding, and the animals were recovered.

### Statistical analyses

Statistics within the main text are presented as mean ± SD [minimum to maximum]. Statistical analyses were performed using GraphPad Prism 10.5.0 (GraphPad Software, Boston), with the specific tests used outlined within the text. Postexperimental power analyses were performed using G*Power 3.1 (Heinrich Heine Universität, Düsseldorf).

## Data Availability

The data supporting the findings of this study are available within the article. Further requests can be made to the corresponding author.

## References

[1] Siegel RL, Giaquinto AN, Jemal A. Cancer statistics, 2024. CA Cancer J Clin. 2024;74(1):12–49. 38230766 10.3322/caac.21820

[2] Park W, Chawla A, O’Reilly EM. Pancreatic cancer: A review. JAMA. 2021;326(9):851–862. 34547082 10.1001/jama.2021.13027 PMC9363152

[3] Orth M, Metzger P, Gerum S, Mayerle J, Schneider G, Belka C, Schnurr M, Lauber K. Pancreatic ductal adenocarcinoma: Biological hallmarks, current status, and future perspectives of combined modality treatment approaches. Radiat Oncol. 2019;14(1):141. 31395068 10.1186/s13014-019-1345-6 PMC6688256

[4] Jemal A, Siegel R, Ward E, Hao Y, Xu J, Thun MJ. Cancer statistics, 2009. CA Cancer J Clin. 2009;59(4):225–249. 19474385 10.3322/caac.20006

[5] Ho WJ, Jaffee EM, Zheng L. The tumour microenvironment in pancreatic cancer—Clinical challenges and opportunities. Nat Rev Clin Oncol. 2020;17(9):527–540. 32398706 10.1038/s41571-020-0363-5 PMC7442729

[6] Gielchinsky I, Lev-Cohain N. Focal irreversible electroporation for localized prostate cancer—Oncological and safety outcomes using mpMRI and transperineal biopsy follow-up. Res Rep Urol. 2023;15:27–35. 36714797 10.2147/RRU.S393243 PMC9880010

[7] Prabhakar P, Avudaiappan AP, Sandman M, Eldefrawy A, Caso J, Narayanan G, Manoharan M. Irreversible electroporation as a focal therapy for localized prostate cancer: A systematic review. Indian J Urol. 2024;40(1):6–16. 38314081 10.4103/iju.iju_370_23 PMC10836445

[8] Onik G, Mikus P, Rubinsky B. Irreversible electroporation: Implications for prostate ablation. Technol Cancer Res Treat. 2007;6(4):295–300. 17668936 10.1177/153303460700600405

[9] Pech M, Janitzky A, Wendler JJ, Strang C, Blaschke S, Dudeck O, Ricke J, Liehr U-B. Irreversible electroporation of renal cell carcinoma: A first-in-man phase I clinical study. Cardiovasc Intervent Radiol. 2011;34(1):132–138. 20711837 10.1007/s00270-010-9964-1

[10] Deodhar A, Monette S, Single GW Jr, Hamilton WC Jr, Thornton R, Maybody M, Coleman JA, Solomon SB. Renal tissue ablation with irreversible electroporation: Preliminary results in a porcine model. Urology. 2011;77(3):754–760. 21111458 10.1016/j.urology.2010.08.036

[11] Min Wah T, Lenton J, Smith J, Bassett P, Jagdev S, Ralph C, Vasudev N, Bhattarai S, Kimuli M, Cartledge J. Irreversible electroporation (IRE) in renal cell carcinoma (RCC): A mid-term clinical experience. Eur Radiol. 2021;31(10):7491–7499. 33825033 10.1007/s00330-021-07846-5 PMC8023551

[12] Narayanan G, Koethe Y, Gentile N. Irreversible electroporation of the hepatobiliary system: Current utilization and future avenues. Medicina. 2024;60(2):251. 38399539 10.3390/medicina60020251 PMC10890312

[13] Spiliopoulos S, Reppas L, Filippiadis D, Delvecchio A, Conticchio M, Memeo R, Inchingolo R. Irreversible electroporation for the management of pancreatic cancer: Current data and future directions. World J Gastroenterol. 2023;29(2):223–231. 36687122 10.3748/wjg.v29.i2.223 PMC9846938

[14] Stephens K, Philips PP, Egger ME, Scoggins CR, McMasters KM, Martin RCG II. Multi-institutional review of adverse events associated with irreversible electroporation in the treatment of locally advanced pancreatic cancer. Surgery. 2024;175(3):704–711. 37852831 10.1016/j.surg.2023.08.042

[15] Jacobs E, Rubinsky B, Davalos R. Pulsed field ablation in medicine: Irreversible electroporation and electropermeabilization theory and applications. Radiol Oncol. 2025;59(1):1–22. 40014783 10.2478/raon-2025-0011 PMC11867574

[16] Davalos RV, Mir ILM, Rubinsky B. Tissue ablation with irreversible electroporation. Ann Biomed Eng. 2005;33(2):223–231. 15771276 10.1007/s10439-005-8981-8

[17] Weaver JC, Chizmadzhev YA. Theory of electroporation: A review. Bioelectrochem Bioenerg. 1996;41(2):135–160.

[18] Neumann E, Schaefer-Ridder M, Wang Y, Hofschneider PH. Gene transfer into mouse lyoma cells by electroporation in high electric fields. EMBO J. 1982;1(7):841–845. 6329708 10.1002/j.1460-2075.1982.tb01257.x PMC553119

[19] Neumann E, Rosenheck K. Permeability changes induced by electric impulses in vesicular membranes. J Membr Biol. 1972;10(3):279–290. 4667921 10.1007/BF01867861

[20] Batista Napotnik T, Polajžer T, Miklavčič D. Cell death due to electroporation—A review. Bioelectrochemistry. 2021;141:107871. 34147013 10.1016/j.bioelechem.2021.107871

[21] Al-Sakere B, Bernat C, Andre F, Opolon P, Davalos R, Mir L. A study of the immunological response to tumor ablation with irreversible electroporation. Technol Cancer Res Treat. 2007;6(4):301–305. 17668937 10.1177/153303460700600406

[22] Bertacchini C, Margotti PM, Bergamini E, Lodi A, Ronchetti M, Cadossi R. Design of an irreversible electroporation system for clinical use. Technol Cancer Res Treat. 2007;6(4):313–320. 17668939 10.1177/153303460700600408

[23] Sorokin I, Canvasser N, Johnson B, Lucas E, Cadeddu JA. Irreversible electroporation for renal ablation does not cause significant injury to adjacent ureter or bowel in a porcine model. J Endourol. 2021;35(6):873–877. 33198480 10.1089/end.2020.0856

[24] Ueshima E, Schattner M, Mendelsohn R, Gerdes H, Monette S, Takaki H, Durack JC, Solomon SB, Srimathveeravalli G. Transmural ablation of the normal porcine common bile duct with catheter-directed irreversible electroporation is feasible and does not impact duct patency. Gastrointest Endosc. 2018;87(1):300.e1–300.e6. 10.1016/j.gie.2017.05.004 PMC5681441 28501593

[25] Maor E, Ivorra A, Leor J, Rubinsky B. The effect of irreversible electroporation on blood vessels. Technol Cancer Res Treat. 2007;6(4):307–312. 17668938 10.1177/153303460700600407

[26] Narayanan G, Bhatia S, Echenique A, Suthar R, Barbery K, Yrizarry J. Vessel patency post irreversible electroporation. Cardiovasc Intervent Radiol. 2014;37(6):1523–1529. 25212418 10.1007/s00270-014-0988-9

[27] Li W, Fan Q, Ji Z, Qiu X, Li Z. The effects of irreversible electroporation (IRE) on nerves. PLOS ONE. 2011;6(4): Article e18831. 21533143 10.1371/journal.pone.0018831 PMC3077412

[28] Zhang K, Teoh J, Laguna P, Dominguez-Escrig J, Barret E, Ramon-Borja JC, Muir G, Bohr J, de Reijke TM, Gómez PP, et al. Effect of focal vs extended irreversible electroporation for the ablation of localized low- or intermediate-risk prostate cancer on early oncological control: A randomized clinical trial. JAMA Surg. 2023;158(4):343–349. 36723911 10.1001/jamasurg.2022.7516 PMC10099059

[29] Månsson C, Brahmstaedt R, Nygren P, Nilsson A, Urdzik J, Karlson BM. Percutaneous irreversible electroporation as first-line treatment of locally advanced pancreatic cancer. Anticancer Res. 2019;39(5):2509–2512. 31092446 10.21873/anticanres.13371

[30] He C, Wang J, Sun S, Zhang Y, Li S. Immunomodulatory effect after irreversible electroporation in patients with locally advanced pancreatic cancer. J Oncol. 2019;2019:9346017. 31214261 10.1155/2019/9346017 PMC6535893

[31] Pandit H, Hong YK, Li Y, Rostas J, Pulliam Z, Li SP, Martin RCG. Evaluating the regulatory immunomodulation effect of irreversible electroporation (IRE) in pancreatic adenocarcinoma. Ann Surg Oncol. 2019;26(3):800–806. 10.1245/s10434-018-07144-3 30610562

[32] He C, Huang X, Zhang Y, Cai Z, Lin X, Li S. Comparison of survival between irreversible electroporation followed by chemotherapy and chemotherapy alone for locally advanced pancreatic cancer. Front Oncol. 2020;10:6. 32038984 10.3389/fonc.2020.00006 PMC6987260

[33] Geboers B, Timmer FEF, Ruarus AH, Pouw JEE, Schouten EAC, Bakker J, Puijk RS, Nieuwenhuizen S, Dijkstra M, van den Tol MP, et al. Irreversible electroporation and nivolumab combined with intratumoral administration of a toll-like receptor ligand, as a means of in vivo vaccination for metastatic pancreatic ductal adenocarcinoma (PANFIRE-III). A phase-I study protocol. Cancers (Basel). 2021;13(15):3902. 34359801 10.3390/cancers13153902 PMC8345515

[34] Ruarus AH, Vroomen LGPH, Geboers B, van Veldhuisen E, Puijk RS, Nieuwenhuizen S, Besselink MG, Zonderhuis BM, Kazemier G, de Gruijl TD, et al. Percutaneous irreversible electroporation in locally advanced and recurrent pancreatic cancer (PANFIRE-2): A multicenter, prospective, single-arm, phase II study. Radiology. 2020;294(1):212–220. 31687922 10.1148/radiol.2019191109

[35] Narayanan G, Hosein PJ, Arora G, Barbery KJ, Froud T, Livingstone AS, Franceschi D, Rocha Lima CM, Yrizarry J. Percutaneous irreversible electroporation for downstaging and control of unresectable pancreatic adenocarcinoma. J Vasc Interv Radiol. 2012;23(12):1613–1621. 23177107 10.1016/j.jvir.2012.09.012

[36] Martin RCG, Kwon D, Chalikonda S, Sellers M, Kotz E, Scoggins C, McMasters KM, Watkins K. Treatment of 200 locally advanced (stage III) pancreatic adenocarcinoma patients with irreversible electroporation safety and efficacy. Ann Surg. 2015;262(3):486–492. 26258317 10.1097/SLA.0000000000001441

[37] Geboers B, van der Lei S, Kloppenborg LTE, Boon RM, Timmer FEF, Puijk RS, de Vries JJ, Scheffer HJ, Meijerink MR. Transcatheter CT arteriography-guided irreversible electroporation of locally advanced pancreatic adenocarcinoma: A pictorial essay. J Med Imaging Radiat Oncol. 2023;67(4):428–434. 37186494 10.1111/1754-9485.13535

[38] Scheffer HJ, Vroomen LGPH, De Jong MC, Melenhorst MCAM, Zonderhuis BM, Daams F, Vogel JA, Besselink MGH, van Kuijk C, Witvliet J, et al. Ablation of locally advanced pancreatic cancer with percutaneous irreversible electroporation: Results of the phase I/II PANFIRE study. Radiology. 2017;282(2):585–597. 27604035 10.1148/radiol.2016152835

[39] Geboers B, Scheltema MJ, Jung J, Bakker J, Timmer FEF, Cerutti X, Katelaris A, Doan P, Gondoputro W, Blazevski A, et al. Irreversible electroporation of localised prostate cancer downregulates immune suppression and induces systemic anti-tumour T-cell activation—IRE-IMMUNO study. BJU Int. 2025;135(2):319–328. 39101639 10.1111/bju.16496 PMC11745989

[40] Liu S, Qin Z, Xu J, Zeng J, Chen J, Niu L, Xu M. Irreversible electroporation combined with chemotherapy for unresectable pancreatic carcinoma: A prospective cohort study. Onco Targets Ther. 2019;12:1341–1350. 30863100 10.2147/OTT.S186721 PMC6388995

[41] Sharma A, Jasrotia S, Kumar A. Effects of chemotherapy on the immune system: Implications for cancer treatment and patient outcomes. Naunyn Schmiedebergs Arch Pharmacol. 2024;397(5):2551–2566. 37906273 10.1007/s00210-023-02781-2

[42] Zhao J, Wen X, Tian L, Li T, Xu C, Wen X, Melancon MP, Gupta S, Shen B, Peng W, et al. Irreversible electroporation reverses resistance to immune checkpoint blockade in pancreatic cancer. Nat Commun. 2019;10(1):899. 30796212 10.1038/s41467-019-08782-1 PMC6385305

[43] Ma Y, Xing Y, Li H, Yuan T, Liang B, Li R, Li J, Li Z, Li S, Niu L. Irreversible electroporation combined with chemotherapy and PD-1/PD-L1 blockade enhanced antitumor immunity for locally advanced pancreatic cancer. Front Immunol. 2023;14:1193040. 37691923 10.3389/fimmu.2023.1193040 PMC10485610

[44] Burbach BJ, O’Flanagan SD, Shao Q, Young KM, Slaughter JR, Rollins MR, Street TJL, Granger VE, Beura LK, Azarin SM, et al. Irreversible electroporation augments checkpoint immunotherapy in prostate cancer and promotes tumor antigen-specific tissue-resident memory CD8+ T cells. Nat Commun. 2021;12(1):3862. 34162858 10.1038/s41467-021-24132-6 PMC8222297

[45] He C, Sun S, Zhang Y, Li S. Irreversible electroporation plus anti-PD-1 antibody versus irreversible electroporation alone for patients with locally advanced pancreatic cancer. J Inflamm Res. 2021;14:4795–4807. 34584438 10.2147/JIR.S331023 PMC8464362

[46] Dhatchinamoorthy K, Colbert JD, Rock KL. Cancer immune evasion through loss of MHC class I antigen presentation. Front Immunol. 2021;12:636568. 33767702 10.3389/fimmu.2021.636568 PMC7986854

[47] Cornel AM, Mimpen IL, Nierkens S. MHC class I downregulation in cancer: Underlying mechanisms and potential targets for cancer immunotherapy. Cancers. 2020;12(7):1760. 32630675 10.3390/cancers12071760 PMC7409324

[48] Fischer JW, Bhattarai N. CAR-T cell therapy: Mechanism, management, and mitigation of inflammatory toxicities. Front Immunol. 2021;12:693016. 34220853 10.3389/fimmu.2021.693016 PMC8250150

[49] Chen P-H, Lipschitz M, Weirather JL, Jacobson C, Armand P, Wright K, Hodi FS, Roberts ZJ, Sievers SA, Rossi J, et al. Activation of CAR and non-CAR T cells within the tumor microenvironment following CAR T cell therapy. JCI Insight. 2020;5(12): Article e134612. 32484797 10.1172/jci.insight.134612 PMC7406247

[50] Gu R, Shen J, Zhang J, Mao J, Ye Q. Revolutionizing autoimmune kidney disease treatment with chimeric antigen receptor-T cell therapy. Research. 2025;8:0712. 40405911 10.34133/research.0712 PMC12095914

[51] Liu L, Bi E, Ma X, Xiong W, Qian J, Ye L, Su P, Wang Q, Xiao L, Yang M, et al. Enhanced CAR-T activity against established tumors by polarizing human T cells to secrete interleukin-9. Nat Commun. 2020;11(1):5902. 33214555 10.1038/s41467-020-19672-2 PMC7677397

[52] Miller IC, Zamat A, Sun L-K, Phuengkham H, Harris AM, Gamboa L, Yang J, Murad JP, Priceman SJ, Kwong GA. Enhanced intratumoural activity of CAR T cells engineered to produce immunomodulators under photothermal control. Nat Biomed Eng. 2021;5(11):1348–1359. 34385695 10.1038/s41551-021-00781-2 PMC8791016

[53] Thomas S, Abken H. CAR T cell therapy becomes CHIC: “Cytokine help intensified CAR” T cells. Front Immunol. 2023;13:1090959. 36700225 10.3389/fimmu.2022.1090959 PMC9869021

[54] Srivastava S, Riddell SR. Engineering CAR-T cells: Design concepts. Trends Immunol. 2015;36(8):494–502. 26169254 10.1016/j.it.2015.06.004 PMC4746114

[55] Sterner RC, Sterner RM. CAR-T cell therapy: Current limitations and potential strategies. Blood Cancer J. 2021;11(4):69. 33824268 10.1038/s41408-021-00459-7 PMC8024391

[56] Tan JY, Low MH, Chen Y, Lim FLWI. CAR T cell therapy in hematological malignancies: Implications of the tumor microenvironment and biomarkers on efficacy and toxicity. Int J Mol Sci. 2022;23(13):6931. 35805933 10.3390/ijms23136931 PMC9266637

[57] Woeste MR, Wilson KD, Kruse EJ, Weiss MJ, Christein JD, White RR, Martin RCG II. Optimizing patient selection for irreversible electroporation of locally advanced pancreatic cancer: Analyses of survival. Front Oncol. 2022;11:817220. 35096621 10.3389/fonc.2021.817220 PMC8793779

[58] Neal RE II, Rossmeisl JH, Robertson JL, Arena CB, Davis EM, Singh RN, Stallings J, Davalos RV. Improved local and systemic anti-tumor efficacy for irreversible electroporation in immunocompetent versus immunodeficient mice. PLOS ONE. 2013;8(5): Article e64559. 23717630 10.1371/journal.pone.0064559 PMC3663742

[59] Ong DY, How GY, Pua U. Irreversible electroporation of the pancreas—A decade on. J Interv Med. 2022;6(1):10–13. 37180371 10.1016/j.jimed.2022.10.001 PMC10167507

[60] Imran KM, Brock RM, Beitel-White N, Powar M, Orr K, Aycock KN, Alinezhadbalalami N, Salameh ZS, Eversole P, Tintera B, et al. Irreversible electroporation promotes a pro-inflammatory tumor microenvironment and anti-tumor immunity in a mouse pancreatic cancer model. Front Immunol. 2024;15:1352821. 38711517 10.3389/fimmu.2024.1352821 PMC11070574

[61] Esparza S, Jacobs E, Hammel JH, Michelhaugh SK, Alinezhadbalalami N, Nagai-Singer M, Imran KM, Davalos RV, Allen IC, Verbridge SS, et al. Transient lymphatic remodeling follows sub-ablative high-frequency irreversible electroporation therapy in a 4T1 murine model. Ann Biomed Eng. 2025;53:1148–1164. 39998766 10.1007/s10439-024-03674-y PMC12006248

[62] Jacobs EJ, Arroyo JP, Powar M, Santos PP, Allen I, Davalos R. Power-driven electroporation is predictive of treatment outcome in a conductivity-independent manner. BME Front. 2025;6:0169. 40801007 10.34133/bmef.0169 PMC12343028

[63] Scuderi M, Dermol-Černe J, Amaral da Silva C, Muralidharan A, Boukany P, Rems L. Models of electroporation and the associated transmembrane molecular transport should be revisited. Bioelectromistry. 2022;147:108216. 10.1016/j.bioelechem.2022.108216 35932533

[64] Rems L, Kasimova MA, Testa I, Delemotte L. Pulsed electric fields can create pores in the voltage sensors of voltage-gated ion channels. Biophys J. 2020;119(1):190–205. 32559411 10.1016/j.bpj.2020.05.030 PMC7335976

[65] Kotnik T, Rems L, Tarek M, Miklavčič D. Membrane electroporation and electropermeabilization: Mechanisms and models. Annu Rev Biophys. 2019;48:63–91. 30786231 10.1146/annurev-biophys-052118-115451

[66] Rems L, Viano M, Kasimova MA, Miklavčič D, Tarek M. The contribution of lipid peroxidation to membrane permeability in electropermeabilization: A molecular dynamics study. Bioelectrochemistry. 2019;125:46–57. 30265863 10.1016/j.bioelechem.2018.07.018

[67] Rems L, Tang X, Zhao F, Pérez-Conesa S, Testa I, Delemotte L. Identification of electroporation sites in the complex lipid organization of the plasma membrane. eLife. 2022;11: Article e74773. 35195069 10.7554/eLife.74773 PMC8912918

[68] Beatty GL, O’Hara MH, Lacey SF, Torigian DA, Nazimuddin F, Chen F, Kulikovskaya IM, Soulen MC, Garvey MM, Nelson AM, et al. Activity of mesothelin-specific chimeric antigen receptor T cells against pancreatic carcinoma metastases in a phase 1 trial. Gastroenterology. 2018;155(1):29–32. 29567081 10.1053/j.gastro.2018.03.029 PMC6035088

[69] Zhai X, Mao L, Wu M, Liu J, Yu S. Challenges of anti-mesothelin CAR-T-cell therapy. Cancers. 2023;15(5):1357. 36900151 10.3390/cancers15051357 PMC10000068

[70] He J, Zhang Z, Lv S, Liu X, Cui L, Jiang D, Zhang Q, Li L, Qin W, Jin H, et al. Engineered CAR T cells targeting mesothelin by piggyBac transposon system for the treatment of pancreatic cancer. Cell Immunol. 2018;329:31–40. 29859625 10.1016/j.cellimm.2018.04.007

[71] Fierle JK, Abram-Saliba J, Atsaves V, Brioschi M, de Tiani M, Reichenbach P, Irving M, Coukos G, Dunn SM. A cell-based phenotypic library selection and screening approach for the de novo discovery of novel functional chimeric antigen receptors. Sci Rep. 2022;12(1):1136. 35064152 10.1038/s41598-022-05058-5 PMC8782825

[72] Tatzel K, Kuroki L, Dmitriev I, Kashentseva E, Curiel DT, Goedegebuure SP, Powell MA, Mutch DG, Hawkins WG, Spitzer D. Membrane-proximal TRAIL species are incapable of inducing short circuit apoptosis signaling: Implications for drug development and basic cytokine biology. Sci Rep. 2016;6(1): Article 22661. 26935795 10.1038/srep22661 PMC4776141

[73] Zurowski D, Patel S, Hui D, Ka M, Hernandez C, Love AC, Lin B, Moore A, Chan LL-Y. High-throughput method to analyze the cytotoxicity of CAR-T cells in a 3D tumor spheroid model using image cytometry. SLAS Discov. 2023;28(3):65–72. 36758833 10.1016/j.slasd.2023.01.008

[74] Ma C, Peng Y, Li H, Chen W. Organ-on-a-chip: A new paradigm for drug development. Trends Pharmacol Sci. 2021;42(2):119–133. 33341248 10.1016/j.tips.2020.11.009 PMC7990030

[75] Geraili A, Jafari P, Hassani MS, Araghi BH, Mohammadi MH, Ghafari AM, Tamrin SH, Modarres HP, Kolahchi AR, Ahadian S, et al. Controlling differentiation of stem cells for developing personalized organ-on-chip platforms. Adv Healthc Mater. 2018;7(2): 10.1002/adhm.201700426. 10.1002/adhm.201700426 28910516

[76] Coluccio ML, Perozziello G, Malara N, Parrotta E, Zhang P, Gentile F, Limongi T, Raj PM, Cuda G, Candeloro P, et al. Microfluidic platforms for cell cultures and investigations. Microelectron Eng. 2019;208:14–28.

[77] Cukierman E, Pankov R, Stevens DR, Yamada KM. Taking cell-matrix adhesions to the third dimension. Science. 2001;294(5547):1708–1712. 11721053 10.1126/science.1064829

[78] Zanoni M, Piccinini F, Arienti C, Zamagni A, Santi S, Polico R, Bevilacqua A, Tesei A. 3D tumor spheroid models for *in vitro* therapeutic screening: A systematic approach to enhance the biological relevance of data obtained. Sci Rep. 2016;6:19103. 26752500 10.1038/srep19103 PMC4707510

[79] Achilli T-M, Meyer J, Morgan JR. Advances in the formation, use and understanding of multi-cellular spheroids. Expert Opin Biol Ther. 2012;12(10):1347–1360. 22784238 10.1517/14712598.2012.707181 PMC4295205

[80] Costa EC, Moreira AF, de Melo-Diogo D, Gaspar VM, Carvalho MP, Correia IJ. 3D tumor spheroids: An overview on the tools and techniques used for their analysis. Biotechnol Adv. 2016;34(8):1427–1441. 27845258 10.1016/j.biotechadv.2016.11.002

[81] Carvalho MR, Lima D, Reis RL, Oliveira JM, Correlo VM. Anti-cancer drug validation: The contribution of tissue engineered models. Stem Cell Rev Rep. 2017;13(3):347–363. 28233276 10.1007/s12015-017-9720-x

[82] Si X, Xiao L, Brown CE, Wang D. Preclinical evaluation of CAR T cell function: In vitro and in vivo models. Int J Mol Sci. 2022;23(6):3154. 35328572 10.3390/ijms23063154 PMC8955360

[83] Mai S, Hodges A, Chen H-M, Zhang J, Wang Y-L, Liu Y, Nakatsu F, Wang X, Fang J, Xu Y, et al. LILRB3 modulates acute myeloid leukemia progression and acts as an effective target for CAR T-cell therapy. Cancer Res. 2023;83(24):4047–4062. 38098451 10.1158/0008-5472.CAN-22-2483 PMC11932437

[84] Liu C, Qi T, Milner JJ, Lu Y, Cao Y. Speed and location both matter: Antigen stimulus dynamics controls CAR-T cell response. Front Immunol. 2021;12:748768. 34691062 10.3389/fimmu.2021.748768 PMC8531752

[85] Zheng N, Fang J, Xue G, Wang Z, Li X, Zhou M, Jin G, Rahman MM, McFadden G, Lu Y. Induction of tumor cell autosis by myxoma virus-infected CAR-T and TCR-T cells to overcome primary and acquired resistance. Cancer Cell. 2022;40(9):973–985.e7. 36027915 10.1016/j.ccell.2022.08.001 PMC9489043

[86] Xue G, Zheng N, Fang J, Jin G, Li X, Dotti G, Yi Q, Lu Y. Adoptive cell therapy with tumor-specific Th9 cells induces viral mimicry to eliminate antigen-loss-variant tumor cells. Cancer Cell. 2021;39(12):1610–1622.e9. 34678150 10.1016/j.ccell.2021.09.011 PMC8678313

[87] Eremenko E, Taylor ZV, Khand B, Zaccai S, Porgador A, Monsonego A. An optimized protocol for the retroviral transduction of mouse CD4 T cells. STAR Protoc. 2021;2(3):100719. 34401785 10.1016/j.xpro.2021.100719 PMC8353356

[88] Li Y, Kurlander RJ. Comparison of anti-CD3 and anti-CD28-coated beads with soluble anti-CD3 for expanding human T cells: Differing impact on CD8 T cell phenotype and responsiveness to restimulation. J Transl Med. 2010;8:104. 20977748 10.1186/1479-5876-8-104 PMC2987859

[89] Duncan BB, Dunbar CE, Ishii K. Applying a clinical lens to animal models of CAR-T cell therapies. Mol Ther Methods Clin Dev. 2022;27:17–31. 36156878 10.1016/j.omtm.2022.08.008 PMC9478925

[90] Lynn RC, Powell DJ Jr. Strain-dependent lethal toxicity in NKG2D ligand-targeted CAR T-cell therapy. Mol Ther. 2015;23(10):1559–1561. 26442803 10.1038/mt.2015.162 PMC4817918

[91] VanSeggelen H, Hammill JA, Dvorkin-Gheva A, Tantalo DGM, Kwiecien JM, Denisova GF, Rabinovich B, Wan Y, Bramson JL. T cells engineered with chimeric antigen receptors targeting NKG2D ligands display lethal toxicity in mice. Mol Ther. 2015;23(10):1600–1610. 26122933 10.1038/mt.2015.119 PMC4817919

[92] Mercadal B, Beitel-White N, Aycock KN, Castellví Q, Davalos RV, Ivorra A. Dynamics of cell death after conventional IRE and H-FIRE treatments. Ann Biomed Eng. 2020;48(5):1451–1462. 32026232 10.1007/s10439-020-02462-8 PMC7154019

[93] Ye C-F, Di Wu J, Li L-R, Sun S-G, Wang Y-G, Jiang T-A, Long X, Zhao J. Co-inhibition of RAGE and TLR4 sensitizes pancreatic cancer to irreversible electroporation in mice by disrupting autophagy. Acta Pharmacol Sin. 2025;46(6):1757–1771. 39953172 10.1038/s41401-025-01487-w PMC12098883

[94] Alnaggar M, Lin M, Mesmar A, Liang S, Qaid A, Xu K, Chen J, Niu L, Yin Z. Allogenic natural killer cell immunotherapy combined with irreversible electroporation for stage IV hepatocellular carcinoma: Survival outcome. Cell Physiol Biochem. 2018;48(5):1882–1893. 30092590 10.1159/000492509

[95] Lin M, Alnaggar M, Liang S, Wang X, Liang Y, Zhang M, Chen J, Niu L, Xu K. An important discovery on combination of irreversible electroporation and allogeneic natural killer cell immunotherapy for unresectable pancreatic cancer. Oncotarget. 2017;8(60):101795–101807. 29254205 10.18632/oncotarget.21974 PMC5731915

[96] Lin M, Zhang X, Liang S, Luo H, Alnaggar M, Liu A, Yin Z, Chen J, Niu L, Jiang Y. Irreversible electroporation plus allogenic Vγ9Vδ2 T cells enhances antitumor effect for locally advanced pancreatic cancer patients. Signal Transduct Target Ther. 2020;5(1):215. 33093457 10.1038/s41392-020-00260-1 PMC7582168

[97] Paul S, Lal G. The molecular mechanism of natural killer cells function and its importance in cancer immunotherapy. Front Immunol. 2017;8:1124. 28955340 10.3389/fimmu.2017.01124 PMC5601256

[98] de Visser KE, Joyce JA. The evolving tumor microenvironment: From cancer initiation to metastatic outgrowth. Cancer Cell. 2023;41(3):374–403. 36917948 10.1016/j.ccell.2023.02.016

[99] Bielamowicz K, Fousek K, Byrd TT, Samaha H, Mukherjee M, Aware N, Wu M-F, Orange JS, Sumazin P, Man T-K, et al. Trivalent CAR T cells overcome interpatient antigenic variability in glioblastoma. Neuro Oncol. 2018;20(4):506–518. 29016929 10.1093/neuonc/nox182 PMC5909636

[100] Ivey JW, Latouche EL, Sano MB, Rossmeisl JH, Davalos RV, Verbridge SS. Targeted cellular ablation based on the morphology of malignant cells. Sci Rep. 2015;5:17157. 26596248 10.1038/srep17157 PMC4657158

[101] Ringel-Scaia VM, Beitel-White N, Lorenzo MF, Brock RM, Huie KE, Coutermarsh-Ott S, Eden K, DK MD, Verbridge SS, Rossmeisl JH Jr, et al. High-frequency irreversible electroporation is an effective tumor ablation strategy that induces immunologic cell death and promotes systemic anti-tumor immunity. EBioMedicine. 2019;44:112–125. 31130474 10.1016/j.ebiom.2019.05.036 PMC6606957

[102] Appelbaum L, Ben-David E, Sosna J, Nissenbaum Y, Goldberg SN. US findings after irreversible electroporation ablation: Radiologic-pathologic correlation. Radiology. 2012;262(1):117–125. 22106355 10.1148/radiol.11110475

[103] Arena CB, Garcia PA, Sano MB, Olson JD, Rogers-Cotrone T, Rossmeisl JH, Davalos RV. Focal blood-brain-barrier disruption with high-frequency pulsed electric fields. Technology. 2014;02(03):206–213.

[104] Garcia PA, Rossmeisl JH, Robertson JL, Olson JD, Johnson AJ, Ellis TL, Davalos RV. 7.0-T magnetic resonance imaging characterization of acute blood-brain-barrier disruption achieved with intracranial irreversible electroporation. PLOS ONE. 2012;7(11): Article e50482. 23226293 10.1371/journal.pone.0050482 PMC3511570

[105] Markelc B, Čemažar M, Serša G. Effects of reversible and irreversible electroporation on endothelial cells and tissue blood flow. In: Miklavčič D, editor. *Handbook of electroporation*. Cham (Switzerland): Springer; 2017. p. 607–620. 10.1007/978-3-319-32886-7_70

[106] Sun Q, Zhou S, Zhao J, Deng C, Teng R, Zhao Y, Chen J, Dong J, Yin M, Bai Y, et al. Engineered T lymphocytes eliminate lung metastases in models of pancreatic cancer. Oncotarget. 2018;9(17):13694–13705. 29568387 10.18632/oncotarget.24122 PMC5862608

[107] Jacobs EJ IV, Graybill PM, Jana A, Agashe A, Nain AS, Davalos RV. Engineering high post-electroporation viabilities and transfection efficiencies for elongated cells on suspended nanofiber networks. Bioelectrochemistry. 2023;152: Article 108415. 37011476 10.1016/j.bioelechem.2023.108415

[108] Haas AR, Golden RJ, Litzky LA, Engels B, Zhao L, Xu F, Taraszka JA, Ramones M, Granda B, Chang W-J, et al. Two cases of severe pulmonary toxicity from highly active mesothelin-directed CAR T cells. Mol Ther. 2023;31(8):2309–2325. 37312454 10.1016/j.ymthe.2023.06.006 PMC10422001

[109] Arroyo JP, Jacobs EJ, Ahmad RN, Amin AJ, Verbridge SS, Davalos RV. Characterization of glioma spheroid viability and metastatic potential following monophasic and biphasic pulsed electric fields. *Bioelectrochemistry*. 2025;165:Article 109005. 10.1016/j.bioelechem.2025.109005 PMC12782275 40398060

